# PATZ1 acts as a tumor suppressor in thyroid cancer via targeting p53-dependent genes involved in EMT and cell migration

**DOI:** 10.18632/oncotarget.2776

**Published:** 2015-01-20

**Authors:** Gennaro Chiappetta, Teresa Valentino, Michela Vitiello, Rosa Pasquinelli, Mario Monaco, Giuseppe Palma, Romina Sepe, Antonio Luciano, Pierlorenzo Pallante, Dario Palmieri, Concetta Aiello, Domenica Rea, Simona Nunzia Losito, Claudio Arra, Alfredo Fusco, Monica Fedele

**Affiliations:** ^1^ Department of Experimental Oncology, Functional Genomic Unit, National Cancer Institute “Fondazione Giovanni Pascale”, IRCCS, 80131 Naples, Italy; ^2^ Institute of Experimental Endocrinology and Oncology (IEOS), National Research Counsil (CNR), 80131 Naples, Italy; ^3^ Animal Facility, National Cancer Institute “Fondazione Giovanni Pascale”, IRCCS, 80131 Naples, Italy; ^4^ Department of Molecular Medicine and Medical Biotechnologies, University of Naples “Federico II”, 80131 Naples, Italy; ^5^ Departments of Molecular Virology, Immunology and Human Genetics, Comprehensive Cancer Center, Ohio State University, Columbus, OH 43210, USA

**Keywords:** thyroid cancer, PATZ1, Epithelial-Mesenchymal Transition, cell migration

## Abstract

PATZ1, a POZ-Zinc finger protein, is emerging as an important regulator of development and cancer, but its cancer-related function as oncogene or tumor-suppressor is still debated. Here, we investigated its possible role in thyroid carcinogenesis. We demonstrated PATZ1 is down-regulated in thyroid carcinomas compared to normal thyroid tissues, with an inverse correlation to the degree of cell differentiation. In fact, PATZ1 expression was significantly further down-regulated in poorly differentiated and anaplastic thyroid cancers compared to the papillary histotype, and it resulted increasingly delocalized from the nucleus to the cytoplasm proceeding from differentiated to undifferentiated thyroid carcinomas. Restoration of PATZ1 expression in three thyroid cancer-derived cell lines, all characterized by fully dedifferentiated cells, significantly inhibited their malignant behaviors, including *in vitro* proliferation, anchorage-independent growth, migration and invasion, as well as *in vivo* tumor growth. Consistent with recent studies showing a role for PATZ1 in the p53 pathway, we showed that ectopic expression of PATZ1 in thyroid cancer cells activates p53-dependent pathways opposing epithelial-mesenchymal transition and cell migration to prevent invasiveness. These results provide insights into a potential tumor-suppressor role of PATZ1 in thyroid cancer progression, and thus may have potential clinical relevance for the prognosis and therapy of thyroid cancer.

## INTRODUCTION

Carcinoma of the thyroid gland is one of the most frequent malignancies of the endocrine system, and its incidence is predicted to become the fourth leading cancer diagnosis by 2030 [1, 2]. Thyroid carcinomas are divided into well-differentiated (WDTCs), poorly differentiated (PDTCs) and anaplastic thyroid carcinomas (ATC) [3, 4]. WDTCs encompass papillary (PTCs) and follicular carcinomas (FTCs). The PTC is the most common thyroid carcinoma (80% of cases). It is often multifocal and tends to metastasize to regional lymph nodes [3]. The FTC is a relatively rare cancer (10% of thyroid cancers) that may develop from a pre-existing benign adenoma (FTA) or directly from the normal tissue. PDTCs and ATCs, can develop *de novo* although many of them arise through the process of stepwise dedifferentiation of PTCs and FTCs [1]. In particular, ATC is a very rare (2–5% of thyroid cancers), highly aggressive and lethal tumor characterized by very undifferentiated cells, mostly insensitive to radiotherapy and conventional chemotherapy [5, 6]. PDTC has an intermediate behavior between WDTC and ATC. Similar to other cancer types, thyroid cancer initiation and progression occurs through gradual accumulation of various genetic and epigenetic alterations. Therefore, according to the theory of sequential progression from WDTC to ATC through PDTC [7], mutations occurring in the early stages of WDTCs are also reported in PDTCs and ATCs [8]. The molecular alteration discriminating ATCs from WDTCs is the inactivation of the p53 tumor suppressor gene. P53 inactivation is observed in almost all ATCs suggesting that p53 deficiency, in association with activating mutations of oncogenes such as RAS and BRAF, drive the high proliferative index and high aggressiveness of these tumors. However, inactivating mutations of p53 observed in several types of human tumors are not frequent in thyroid cancer, but studies on p53 protein expression in a large series of thyroid tumor specimens suggest that, although not mutated, p53 activity may be inhibited in thyroid cancer by other mechanisms [9].

In spite of the progressive knowledge of the molecular mechanisms involved in thyroid transformation, the prognosis of thyroid cancer remains unpredictable and the identification of new biological markers are needed in addition to already known molecules, to correctly stratify patients at risk of recurrence and progression [10].

The POZ/BTB and AT-hook-containing zinc finger protein 1 (PATZ1) is a transcriptional regulatory factor also known as Zinc finger Sarcoma Gene (ZSG), MAZ-Related factor (MAZR) or Zinc Finger Protein 278 (ZNF278/Zfp278). PATZ1 has been demonstrated to regulate, either positively or negatively, the expression of different genes depending on the cellular context [11–17].

Several studies suggest a role for PATZ1 in cancer but its cancer-related function as oncogene or tumor suppressor is still debated. PATZ1 oncogenic role is supported by its overexpression in human malignant neoplasias, including colon and breast tumors [18, 19] and its down-regulation by siRNAs either blocks the growth or induces apoptosis of cell lines derived from colorectal cancer or gliomas, respectively [18, 20]. Similarly, we previously demonstrated that PATZ1 is overexpressed in testicular tumors, but protein localized into the cytoplasm rather than into the nucleus, suggesting a reduction of its transcriptional function [21]. Recently, we showed that PATZ1-knockout mice develop lymphomas and other neoplasias, indicating PATZ1 as a potential tumor-suppressor in lymphomagenesis and likely other tumors [17].

In this study we have analyzed PATZ1 expression and function in human thyroid cancer, identifying a potential tumor suppressor role in this type of cancer, mainly involved in inhibition of epithelial-mesenchymal transition (EMT) and cell migration.

## RESULTS

### PATZ1 is down-regulated and delocalized in thyroid cancer

The expression of *PATZ1* gene was analyzed, by quantitative RT-PCR (qRT-PCR), in human thyroid cancer cell lines and tissues compared to normal thyroids (NT).

**Figure 1:**
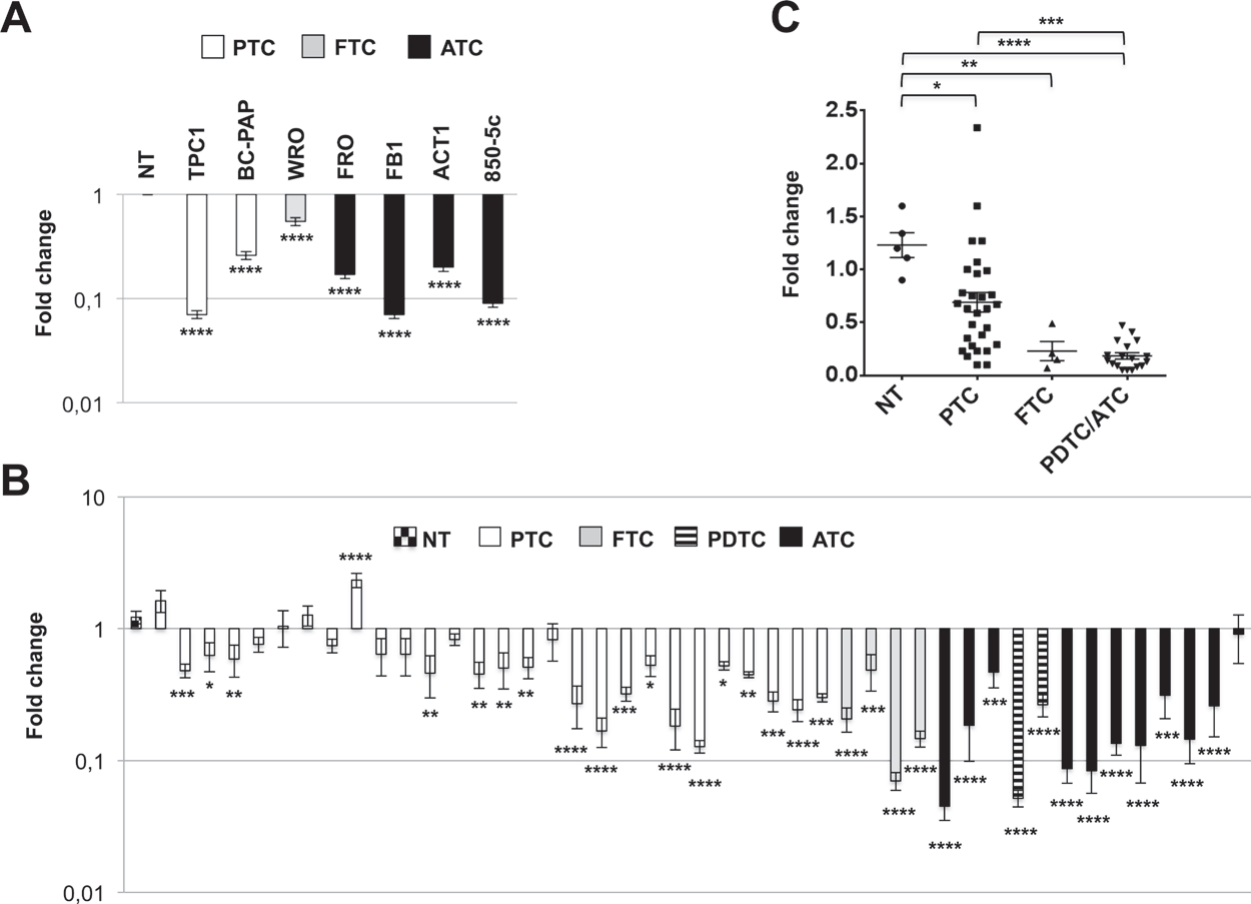
PATZ1 expression in human thyroid cancer cell lines and tissues **(A)** qRT-PCR analysis of *PATZ1* in 2 PTC-derived cell lines (TPC1 and BC-PAP), 1 FTC-derived cell line (WRO) and 4 ATC-derived cell lines (FRO, FB1, ACT1, 850-5c) in comparison with 3 normal thyroid gland tissues, whose mean value of expression was set to 1. Mean values ± SE of triplicate samples compared to each normal control are shown. NT = mean value ± SE of the three normal thyroid tissues used as control. **(B)** qRT-PCR analysis of *PATZ1* in 28 PTCs, 4 FTCs, 2 PDTC and 11 ATCs in comparison with mean value of 5 normal thyroid samples (first lane). Mean values ± SE of two independent experiments for each sample, performed in duplicate, compared to each normal control, which has been set to 1, are shown. All values in A and B are shown in a logarithmic scale and were analyzed by one-way ANOVA followed by Dunnett's multiple comparison test. **(C)** All samples shown in B were grouped for histotype and analyzed by one-way ANOVA followed by Tukey's multiple comparison test. Mean values ± SE are shown. *, *P* < 0.05; **, *P* < 0.01; ***, *P* < 0.001; ****, *P* < 0.0001.

The thyroid cancer cell lines used were derived from papillary (TPC1, BC-PAP), follicular (WRO) and anaplastic (FRO, FB1, ACT1, 850-5c) thyroid carcinomas. As shown in Figure 1A, in all the analyzed cell lines, *PATZ1* expression was significantly reduced compared to normal control, represented by mean value of three normal thyroid tissues.

The analysis of PATZ1 expression on tissue samples, carried out on 5 NTs, 28 PTCs, 4 FTCs, 2 PDTCs and 11 ATCs, showed a significant down-regulation of *PATZ1* in 64% of PTCs, 91% of ATCs and 100% of FTCs and PDTCs (Figure 1B). Indeed, as shown in Figure 1C, the multiple comparison analysis of the results demonstrated that PATZ1 was not only significantly downregulated in PTC (*P* < 0.05), FTC (*P* < 0.01) and PDTC/ATC (*P* < 0.0001) *versus* NT, but also it was significantly further down-regulated in PDTC/ATC *versus* PTC (*P* < 0.001).

These results indicate that PATZ1 expression is negatively associated with thyroid cancer progression, suggesting it could play a tumor suppressor role in thyroid cancer, mainly involved in the late stages of carcinogenesis.

**Figure 2:**
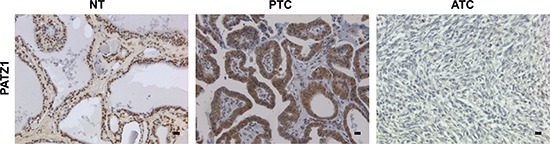
Representative images of PATZ1 staining in normal thyroid (NT), papillary thyroid carcinoma (PTC) and anaplastic thyroid carcinoma (ATC) PATZ1 staining was intense in the nucleus of normal thyroid tissue; it is present also in the cytoplasm of PTC; it was absent in ATC. Scale bars = 100 nm.

Subsequently, we analyzed PATZ1 protein expression and localization by immunohistochemistry (IHC). The analysis was performed on paraffin embedded normal and neoplastic thyroid samples, including 27 NTs, 2 goiters, 11 FTAs, 33 PTCs, 12 FTCs, 5 PDTCs and 18 ATCs. All samples of normal thyroid parenchyma and goiters expressed PATZ1 at a high level in the nucleus, which coincides with the strong PATZ1 staining in all follicles. Conversely, compared to normal samples, PATZ1 expression in the nucleus was found to be weaker in a high percentage of FTAs (73%), PTCs (36%) and FTCs (50%), and weak or completely negative in most PDTCs (100%) and ATCs (83%) (Figure 2 and Table 1). Interestingly, PATZ1 protein showed a progressive displacement from the nucleus to the cytoplasm with a direct correlation with the undifferentiated and malignant phenotype. Indeed, in all NTs (100%, 27/27) and goiters (100%, 2/2) analyzed, PATZ1 was expressed and present only in the nucleus, while in most FTAs (64%), PTCs (82%) and FTCs (100%), PATZ1 protein was partially or completely localized into the cytoplasm. In 60% (3/5) of PDTCs PATZ1 protein was localized only in the cytoplasm as in 11% of ATCs. Moreover, in 20% of PDTCs and in 22% of ATCs, PATZ1 expression was completely absent (Table 1). These results confirmed an inverse correlation between PATZ1 expression and the thyroid malignant phenotype.

**Table 1:** PATZ1 nuclear expression and sub-cellular localization

Hystotype	N.	Nuclear anti-PATZ1 reactivity			PATZ1 sub-cellular localization			
		Negative	Weak	Strong	Nuclear	Nucl/cyt	Cytosol	Negative
**NT**	27	0	6 (22%)	21 (78%)	27 (100%)	0	0	0
**Goiter**	2	0	0	2 (100%)	2 (100%)	0	0	0
**FTA**	11	2 (18%)	8 (73%)	1 (9%)	4 (36%)	6 (54%)	1 (9%)	1 (9%)
**PTC**	33	0	12 (36%)	21 (64%)	6 (18%)	27 (82%)	0	0
**FTC**	12	0	6 (50%)	6 (50%)	0	12 (100%)	0	0
**PDTC**	5	4 (80%)	1 (20%)	0	0	1 (20%)	3 (60%)	1 (20%)
**ATC**	18	6 (33%)	9 (50%)	3 (17%)	7 (39%)	5 (28%)	2 (11%)	4 (22%)

### PATZ1 expression inhibits growth of BC-PAP and FRO cells

**Figure 3:**
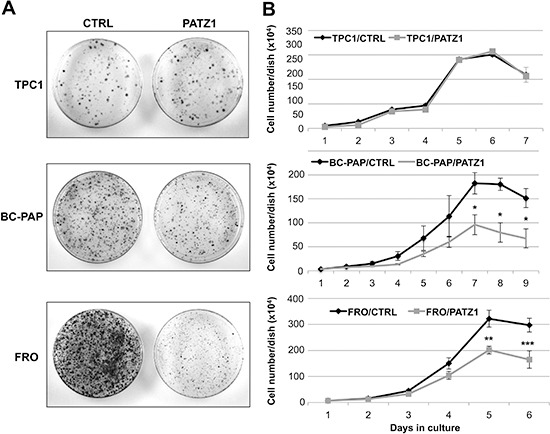
Analysis of cell growth in PATZ1-transfected thyroid cancer cells **(A)** Colony-forming assays in human thyroid cancer cell lines transfected with PATZ1. TPC-1, BC-PAP and FRO cells were transfected with a vector expressing PATZ1 cDNA or its corresponding empty vector. Cells were cultured for 10 days, selected for resistance to G418, and stained with crystal violet. **(B)** Growth curves on different stably expressing PATZ1 cell clones and/or mass populations of TPC1, BC-PAP and FRO cells compared to controls expressing the empty vector. Mean values ± SE of at least three clones for each cell line are reported: For TPC1, clone C-1, parental TPC1 and mock-transfected mass population were used as control, whereas clones PA1, PA5 and PA6 were used as PATZ1-transfected cells; for FRO, clones C-4, C-7 and parental FRO were used as control, whereas clones PA11, PA13 and PA17 were used as PATZ1-transfected cells; for BC-PAP, clone C-1, parental BC-PAP and mock-transfected mass population (mp C-) were used as control, whereas PA2, PA3, PA7, PA10 and PATZ1-transfected mass population (mp PA) were used as PATZ1-transfected cells. PATZ1 expression in each clone or mass population is shown in supplementary Figure S1. *, *P* < 0.05; **, *P* < 0.01; ***, *P* < 0.001.

To determine whether the expression of PATZ1 plays a role in thyroid cancer cell growth, we carried out colony-forming assays in three thyroid cancer cell lines (TPC1, BC-PAP and FRO), transfected with a vector coding for the human PATZ1 variant 4 (HA-PATZ) or the empty vector (pCEFL-HA). As shown in Figure 3A, in both BC-PAP and FRO cells an evident decrease of the colony number was detected in PATZ1 transfected cells compared to controls. Conversely, no appreciable differences were found in TPC1 cells transfected with PATZ1 or the empty vector.

Next, in order to deeply investigate a possible causal role of PATZ1 in thyroid cancer cell proliferation and other thyroid cancer cell functions, we transfected a PATZ1-EGFP-C2 plasmid carrying human PATZ1 variant 4 cDNA, or the empty vector pEGFP-C2 into the three thyroid cancer cell lines already used for the colony assays and selected mass populations and/or cell clones in which PATZ1 expression was stably up-regulated compared to parental cells transfected with the empty vector (supplementary Figure S1).

To confirm the results of the colony assays and deeper investigate the cause of growth inhibition, we performed growth curves and cell viability assays on selected clones of TPC1/PATZ1, BC-PAP/PATZ1 and FRO/PATZ1 compared to their respective controls (Figure 3B). In agreement with the results from the colony assays, the growth rate of TPC-1/PATZ1 clones did not show any difference compared to control. Conversely, BC-PAP/PATZ1 clones, and FRO/PATZ1 clones, showed decreased proliferation capacity, starting to be significant at 7 or 5 days of cell culture, respectively, without differences in trypan blue incorporation (data not shown), compared to control cells.

### PATZ1 expression inhibits cell migration and invasion of TPC-1, BC-PAP and FRO cells

**Figure 4:**
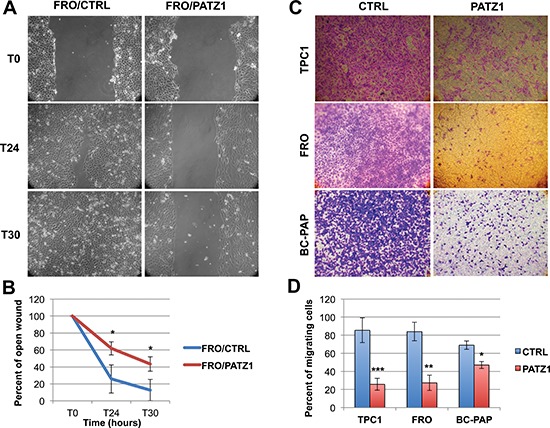
PATZ1 inhibits cellular migration in TPC1, FRO and BC-PAP cells **(A)** Representative images of a wound healing assay in control (CTRL) and PATZ1-expressing FRO cells at 0, 24 h and 30 h after a confluent cell monolayer was wounded. **(B)** Percent of open wound calculated as mean values ± SE of two FRO/CTRL (C-4 and C-7) and four FRO/PATZ1 cell clones (PA2, PA10, PA11 and PA13). **(C)** Representative images of a transwell assay in CTRL and PATZ1-expressing TPC1, FRO and BC-PAP cells. Migrating cells were stained with crystal violet. **(D)** The number of migrating cells was calculated by measuring the percentage of stained cells. Mean values ± SE of at least 3 different clones (C-1, parental TPC1, PA1, PA5 and PA6 for TPC1; C-4, C-7, parental FRO, PA2, PA13, PA16 and PA17, for FRO; mp C-, mp PA, PA2, PA3, PA7 and PA10 for BC-PAP) in 3 independent experiments are reported. PATZ1 expression in each clone or mass population is shown in supplementary Figure S1. *, *P* < 0.05; **, *P* < 0.01; ***, *P* < 0.001.

Next, using a wound-healing assay, we tested the migration capacity of PATZ1-transfectants, showing that it was significantly reduced in FRO/PATZ1 compared with the control cells (Figure 4A–4B). Conversely, no significant differences were observed in BC-PAP and TPC1 cells (data not shown). These data indicate that PATZ1 can inhibit migration of thyroid cancer cells, but also suggest that this role is cell context-dependent. However, the wound-healing assay is particularly suitable for studying the effects of cell-matrix and cell-cell interactions on cell migration, but does not give insights on migration in response to a particular chemical signal, which is usually referred to as chemotaxis. To better investigate this issue we analyzed cell migration across 8-μm membrane pores in response to FBS. At 24 h after seeding, all PATZ1-transfected clones, including TPC1, BC-PAP and FRO cells, migrated less than empty vector control cells (Figure 4C–4D). At this experimental time, influence of PATZ1 in cell proliferation was absent (supplementary Figure S2). Next, we also directly examined the *in vitro* capacity of these cells to invade through a Matrigel-coated membrane, which has been reported to mimic the whole process of invasion, including adhesion to a substrate, dissolution of the extracellular matrix and migration [25]. Using this assay, we observed a decrease in invading capacity of PATZ1-transfected cells compared to empty vector controls, that reached significant levels in FRO and BC-PAP and was close to be significant in TPC1 cells (supplementary Figure S3). All together these results indicate that PATZ1 has a key role in suppressing migration and invasiveness of thyroid cancer cells, but also suggest that this role could involve different aspects of cell migration in different cellular contexts.

### PATZ1 expression inhibits tumorigenicity of FRO cells and induces a mesenchymal-epithelial-like transition

**Figure 5:**
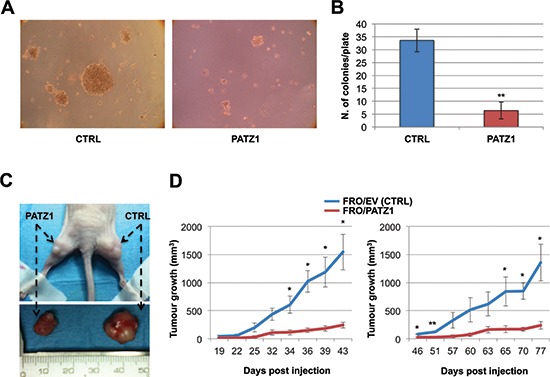
PATZ1 inhibits *in vitro* and *in vivo* tumorigenicity in FRO cells **(A)** Representative images of growth in soft agar of control (CTRL) and PATZ1-expressing FRO cells. **(B)** Colonies larger than background (as observed in normal control cells) were counted after 2 weeks. Mean values ± SE of two controls (C-4 and parental FRO) and three PATZ1-expressing clones (PA13, PA16, PA17) are reported. PATZ1 expression in each clone is shown in supplementary Figure S1. **, *P* < 0.01. **(C)** Representative nude mouse (upper panel) injected with FRO/PATZ1 cells (clone PA13) at the left side and FRO/EV (CTRL) cells (clone C-4) at the right side. Tumors (lower panel) were excised when one of the two contralateral tumors reached the cut-off of 1500 mm^3^. **(D)** Tumor growth curves in cohorts of 3 mice (left panel) and 4 mice (right panel). Mean values ± SE are reported. *, *P* < 0.05; ** *P* < 0.01. FRO/EV = empty vector-transfected FRO cells.

**Figure 6:**
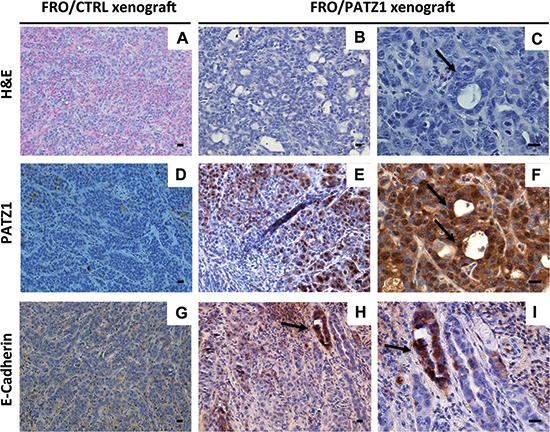
FRO/PATZ1 xenografts showed an epithelial-like phenotype Representative images of tumor tissues derived from nude mice injected with FRO/PATZ1 **(B, C, E, F, H, I)** or control (CTRL) cells **(A, D, G)** from the experiments shown in Figure 5C. Tumors developed from PATZ1-expressing cells showed features of epithelial-like differentiation represented by follicular-like structures (arrows). Consistently, E-cadherin immunostaining revealed a strong positive reaction in such follicular-like structures (H, I). Immunostaining for PATZ1 showed strong expression of PATZ protein in delimited areas including cells with an epithelial-like phenotype (E, F), whereas it was negative in all the other areas of PATZ1 xenografts (E) and in CTRL counterparts (D) Scale bars = 100 nm. H&E = hematoxilin and eosin staining.

To characterize the malignant phenotype of the stable transfectants, we analyzed their ability to grow in soft agar. Only parental and backbone vector-transfected FRO cells were able to form large, progressively growing colonies. In contrast, FRO/PATZ1 transfectants showed a drastic reduction in colony-forming efficiency (Figure 5A–5B). Both TPC1 and BC-PAP cell lines, with or without transfected PATZ1, did not form any colony in soft agar (data not shown). We next investigated the capacity of FRO/PATZ1 and their controls to generate tumor xenografts in nude mice. Tumor growth was observed in 7 out of 7 mice injected with empty vector-transfected FRO (FRO/EV) and 7 out of 7 mice xenografted with FRO/PATZ1 cells. However, size and growth rate of tumors derived from FRO/PATZ1 cells were drastically reduced compared to those of tumors generated by control cells injected into the contralateral leg (Figure 5C–5D). The histopathological analysis of the tumors, excised at the end of their growth observation, revealed that, unlike all FRO/EV-induced tumors, in which tumor tissue was composed of anaplastic cells irregularly arranged in a mass with solid aspects, 4/7 FRO/PATZ1-derived xenografts appeared heterogeneous with some areas displaying a phenotypic switch towards a better organized structure with epithelial-like features, sometimes resembling follicular structures (Figure 6). Interestingly, only in FRO/PATZ1 tumors showing follicular-like structures we observed overexpression of PATZ1, due to residual areas of cells expressing PATZ1, whereas in all the others PATZ1 expression was completely lost, as in tumors originated from control-transfected cells (supplementary Table S1 and Figure S4). It is likely that cells in which PATZ1 expression is lost are able to grow faster *in vivo*, giving rise to tumors that phenocopy the anaplastic tumor from which FRO cells originated. Conversely, PATZ1-expressing cells grow slower and the general tumor growth is likely due to those cells in which expression of PATZ1 was lost, which tend to prevail over those expressing PATZ1. Moreover, consistent with a possible association between PATZ1 expression and mesenchymal-epithelial transition, in tumor areas showing expression of PATZ1 and follicular-like structures we observed positive staining for E-cadherin (Figure 6).

### PATZ1 expression activates the p53 pathway involved in the block of EMT, migration and invasiveness

**Figure 7:**
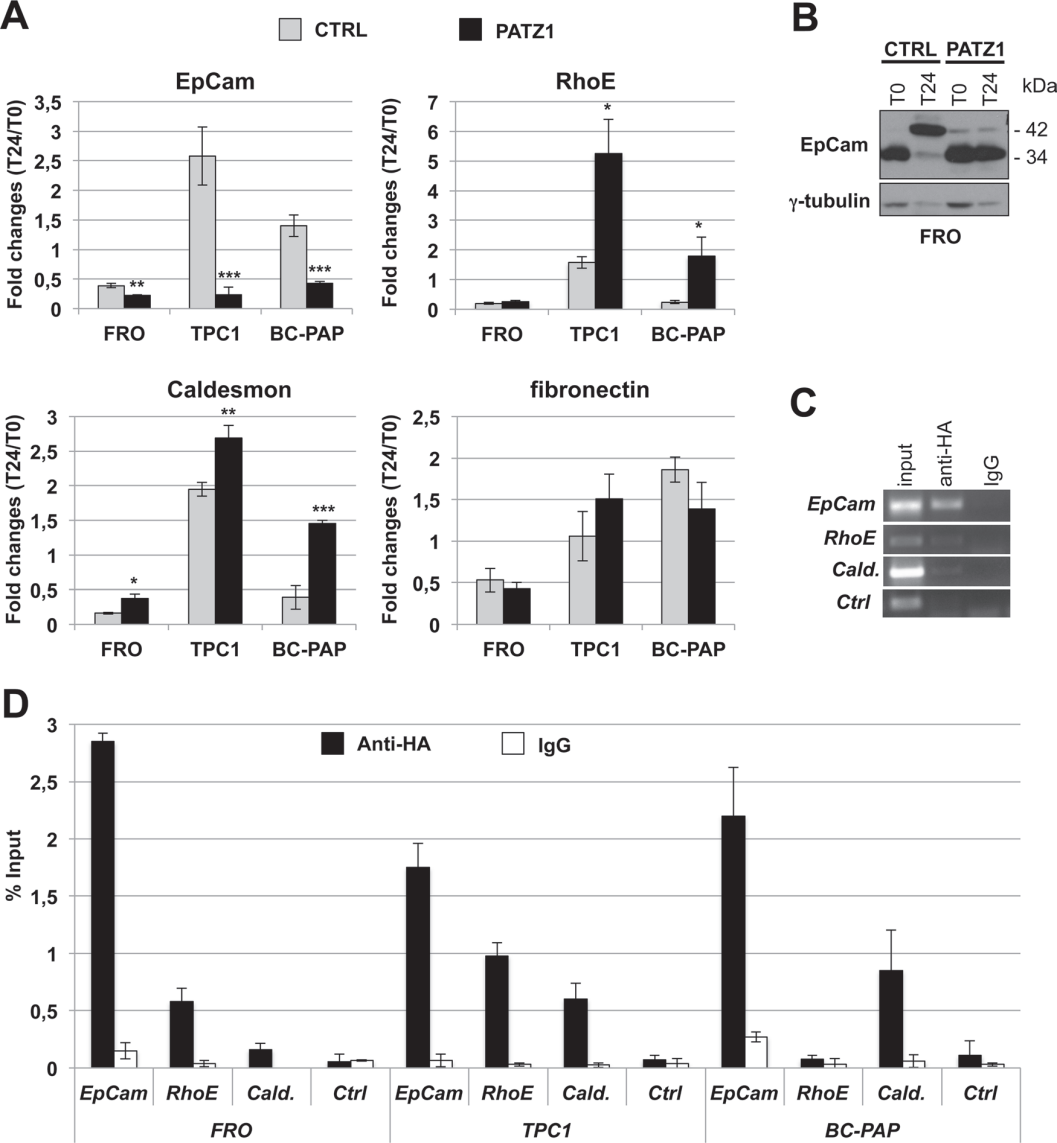
PATZ1 re-introduction affects expression of p53 target genes involved in EMT, migration and invasion **(A)** FRO/PATZ1, TPC1/PATZ1, BC-PAP/PATZ1 transfectants and their respective controls were starved for 48 h (T0) and then stimulated with epidermal growth factor (EGF) for 24 h (T24). qRT-PCR showing expression, measured as fold changes at T24 with respect to T0, of *EpCAM*, *Caldesmon*, *RhoE* and *Fibronectin* genes. Mean values ± SE of two or three independent clones are reported. For TPC1, parental TPC1 and clone C-1 were used as control, whereas clones PA1, PA5 and PA6 were used as PATZ1-transfected cells; for FRO, clones C-4 and parental FRO were used as control, whereas clones PA11 and PA17 were used as PATZ1-transfected cells; for BC-PAP, parental BC-PAP, clone C-1 and mock-transfected mass population (mp C-) were used as control, whereas PA7, PA10 and PATZ1-transfected mass population (mp PA) were used as PATZ1-transfected cells. PATZ1 expression in each clone or mass population is shown in supplementary Figure S1. *, *P* < 0.05; ** *P* < 0.01. **(B)** Western blot analysis of EpCam expression in FRO cells at 0 h (T0) and 24 h (T24) from EGF treatment. Where indicated, CTRL = control; PATZ1 = PATZ1-expressing cells. **(C)** representative ChIP experiments in FRO cells, transiently transfected with HA-PATZ1 and immunoprecipitated with anti-HA Ab, to detect *in vivo* binding of PATZ1 to *EpCam (−218/−65)*, *RhoE (−2239/−2183)* and *Caldesmon (−106/−2)* promoter regions. IgG = not-specific Ab, *Cald*. = Caldesmon, *Ctrl* = *Caldesmon* region *-951/−841*, Input *=* PCR products with genomic DNA without immunoprecipitation. **(D)** Semi-quantitative analysis of ChIP assays on FRO, TPC1 and BC-PAP cells performed by densitometric evaluation of the gels (ImageJ64 software). Results shown are the mean values ± SD of two independent experiments for each gene and cell line, expressed as percentage of PATZ1 immunoprecipitated DNA relative to the Input. IgG and *Ctrl* abbreviations are as in (C).

To gain insight into the molecular mechanisms involved in PATZ1-mediated inhibition of cell migration, we analyzed expression of a panel of genes playing crucial roles in this key stage of metastatic progression. In particular, we focused on genes downstream of p53, because of recent data showing a functional interaction between PATZ1 and p53 [11, 26], analyzing their expression before and after a stimulus to migrate. To this aim, TPC1/PATZ1, FRO/PATZ1, BC-PAP/PATZ1 transfectants and their respective controls were starved for 48 h and then stimulated with epidermal growth factor (EGF) for 24 h. It is known that p53 maintains a transcriptional program to prevent EMT by downregulating genes, such as EpCAM, that inhibit molecules involved in stabilizing cell-cell junctions (such as E-cadherin), or by directly inhibiting components of the adhesive machinery, such as Fibronectin, that are known to contribute to cell motility through the stroma [27]. p53-regulated genes also include molecules involved in inhibition of podosome formation, such as the actin-binding protein Caldesmon, which is up-regulated by p53 [28]. Finally, p53 can also up-regulate molecules that control actin dynamics, such as RhoE and NOTCH, which both converge in the inhibition of cytoskeletal changes accompanying tumor cell migration and invasion [27]. It is noteworthy that *TP53* gene is hypo-fuctioning, but wt in FRO and TPC1 cells [29, 30], whereas it is mutated in BC-PAP cells [30]. As shown in Figure 7A, by (q)RT-PCR, expression of *EpCam* and *Caldesmon* in all three cell lines, and *RhoE* in TPC1 and BC-PAP cells, were significantly changed in PATZ1 expressing clones compared to control cells. Conversely, no changes were observed in *RhoE* and *Fibronectin* gene expression, in FRO and all three cell lines, respectively, between PATZ1-expressing clones and controls. In particular, *EpCam* expression was downregulated about 3-fold in FRO control cells following treatment with EGF, and significantly further downregulated, up to about 5-fold, in FRO clones expressing PATZ1 (Figure 7A); *Caldesmon* and RhoE were up-regulated about 2-fold and 1.5-fold, respectively, in TPC1 control cells following treatment with EGF, and further up-regulated, up to about 3- and 7-fold, respectively, in TPC1 clones expressing PATZ1 (Figure 7A). All together these results suggest that in both FRO and TPC1 cells a partial functional p53 protein, at least on the *EpCam* promoter in FRO cells and on the Caldesmon and RhoE promoters in TPC1 cells, is present but its activity can be enhanced by PATZ1, which appears to cooperate with p53 in opposing to EMT in FRO cells and to cell motility and invasiveness in TPC1 cells.

Conversely, as expected by the presence of a mutant p53 in BC-PAP cells [30], treatment with EGF in these cells resulted in an opposite regulation of the above mentioned genes, including upregulation of *EpCam* and *Fibronectin*, and down-regulation of *Caldesmon* and *RhoE*, with the consequent activation of the EMT and cell migration programs (which are not opposed by a functional p53). Importantly, in BC-PAP/PATZ1 transfectants these functions are partially or totally rescued. In fact, as shown in Figure 7, following EGF treatment, *EpCam* was up-regulated in BC-PAP control cells, whereas it was downregulated in 2 out of 3 PATZ-expressing BC-PAP clones. Similarly, *Caldesmon* and *RhoE* were downregulated in BC-PAP control cells, whereas they were upregulated in PATZ1 transfectants. Notably, *EpCam* resulted up-regulated also in TPC1 control cells following EGF treatment, but this behaviour was completely reverted in TPC1/PATZ1 clones. These results suggest that PATZ1 can activate the p53 pathway opposing EMT, migration and invasiveness also in presence of a mutant p53.

Subsequently, to assess a direct action of PATZ1 on transcription of these genes, we performed ChIP assays to avaluate PATZ1 protein binding to their promoters. Therefore, TPC1, FRO and BC-PAP cells, transiently transfected with a HA-tagged PATZ1 expression vector, were cross-linked and immunoprecipitated with anti-HA or isotype-matched preimmune IgG. Immunoprecipitation of chromatin was then analyzed by qualitative PCR, using primers spanning regions including the binding site for p53 or potential consensus elements for PATZ1. As shown in Figure 7C–7D, PATZ1 binding to *EpCam* and *Caldesmon* genes was detected in all three cell lines, binding to *RhoE* was detected in TPC1 and FRO cells, whereas a distal region on the *Caldesmon* promoter (*Ctrl*) was not amplified in any cell line, strenghtening the specificity of the binding of PATZ1 detected in the ChIP assays.

Only for EpCam, which has been recently suggested to be involved in the development of the aggressive phenotype of ATC [31] and has evoked significant interest as a target in cancer therapy [32] we analyzed protein expression changes in proliferating, starved (T0) and EGF-induced (T24) FRO, TPC1 and BC-PAP cells. Consistent with the work of Okada et al [31], reporting that EpCam protein was expressed only in anaplastic–derived thyroid cancer cell lines, we detected EpCam expression by Western blot only in FRO cells (Figure 7B and data not shown). Interestingly, EpCAM appeared to undergo a post-translational modification following treatment with EGF, as suggested by the uppershift of the protein size. This change appears to be inhibited by the presence of PATZ1 (Figure 7B).

## DISCUSSION

Despite an increasing body of evidences is highlighting PATZ1 as a cancer-related gene [15–21], little is known about its function. A dual role favoring transformation or protecting from it, depending on the cellular context, seems to apply for the PATZ1 protein [11]. However, still few tumors have been analyzed for PATZ1 expression and function. Here we focus on thyroid cancer, one of the most frequent malignancies of the endocrine system, whose mechanisms of transformation are still far from being completely elucidated [1]. We first analyzed a wide panel of thyroid cancer cell lines and tissues, observing that PATZ1 is expressed at significantly lower levels compared to normal thyroid tissues. Moreover, PATZ1 protein is partially or completely delocalized from nucleus to cytoplasm in most of carcinoma samples. Interestingly, PATZ1 downregulation, as well as its cytoplasmic localization, correlates with the acquisition of a less differentiated phenotype, suggesting that PATZ1 loss and cytoplasmic localization could be considered as a valid marker of an undifferentiated, mesenchymal and aggressive phenotype. Notably, we showed that PATZ1 is strongly downregulated in all the thyroid cancer cell lines analyzed. However, despite their different origin, these cell lines have gene expression profiles more closely related to each other than to the *in vivo* differentiated tumors they were derived and have characteristics of fully dedifferentiated cells, close to undifferentiated carcinomas [33].

Therefore, choosing the thyroid cancer cell lines TPC1, BC-PAP and FRO, as cellular models for undifferentiated tumors, we showed that in all of them reintroduction of PATZ1 leads to inhibition of cellular capacity to migrate and invade, supporting a role for PATZ1 in opposing the late steps of thyroid transformation, consisting in the acquisition of a mesenchymal phenotype (EMT), capable to migrate and invade surrounding tissues, thus giving rise to local and distal metastases. Consistent with the involvement of PATZ1 in EMT, it has been recently shown that PATZ1 is part of a group of transcription factors, including proteins already linked to EMT, such as EGR-1, Sp1, Sp2, NME1, CTCF, PLAG1 and WT1, potential regulators of TGF-β1 [34]. PATZ1 reintroduction in BC-PAP and FRO cells also affected cell proliferation, suggesting a possible role for PATZ1 in this cellular function depending on the cellular context. We finally showed that PATZ1 significantly inhibits FRO tumorigenic potential both *in vitro* and *in vivo*. Interestingly, tumors grafted from PATZ1-expressing cells showed some epithelial-like features, including follicular-like structures and E-cadherin expression. Notably, the tumor areas displaying such features were also enriched in PATZ1 expression, supporting the idea that PATZ1 could have a direct role in a mesenchymal to epithelial transition (MET)-like state. Consistently, the majority of the tumors derived from the injection of PATZ1-cells resulted mostly negative for PATZ1 expression and displayed a mesenchymal phenotype similar to those arisen from control cells. Therefore, it is likely that PATZ1 has been negatively selected *in vivo* as a way to allow an EMT phenotype.

EMT is involved in many biological processes including embryonic development, wound-healing and cancer progression [35]. In thyroid cancer it seems to be specifically involved in the development of ATCs [36], but there are evidences of its involvement also in local invasion of PTCs [37]. It is increasingly acknowledged that EMT plays an important role in the metastasis of many types of carcinomas [38, 39] and has been implicated in therapeutic resistance and tumor recurrence [40, 41]. Therefore, the identification of genes able to modulate these cellular processes has a great potential for a targeted cancer therapy. One of the major players opposing to EMT is the tumor suppressor p53 protein, whose loss has been shown to influence motility contributing to the invasive and metastatic potential of cancer cells [27]. In particular, it has been shown that p53 maintains a transcriptional program to prevent EMT and that loss of this suppression may contribute to the induction of an EMT-like phenotype [42, 43].

Recent data, showing that PATZ1 is able to interact with p53 and to directly regulate transcription of p53-regulated genes [11], suggested a possible mechanism by which PATZ1 may be involved in EMT. Consistently, we observed that PATZ1 binds *in vivo* some of the p53-regulated genes involved in preventing EMT, and its overexpression causes changes in their expression changes associated to the stimulation of cell migration by EGF. Importantly, in cells carrying a wild-type *TP53* gene, such as TPC1 and FRO, the resulting effect is a potentiation of the transcriptional program opposing EMT, migration and invasiveness. Conversely, in cells carrying a mutant *TP53* gene, such as BC-PAP, the presence of PATZ is only partially able to activate such program, indicating that PATZ1 is not sufficient to regulate these p53-dependent genes in presence of a mutant p53. Interestingly, PATZ1 expression seems to affect expression of different genes depending on the cellular context: in FRO cells it downregulates *EpCAM*, involved in the inhibition of E-cadherin [27], and upregulates *Caldesmon*, implicated in the inhibition of invadopodia [27]; in TPC1 cells it upregulates *RhoE*, implicated in the inhibition of cytoskeletal changes accompanying tumor cell migration [27], and *Caldesmon*, whereas downregulates *EpCam*; in BC-PAP cells it modulates all three of these genes. The absence of PATZ1 binding to the *RhoE* promoter in BC-PAP cells suggests that a functional p53 gene is required for this binding. However, we cannot exclude that in this cell line, EGF treatment is necessary to induce binding of PATZ1 to this gene. Further axperiments are needed to better elucidate the dynamic of PATZ1 binding to these genes in the different cell lines and in relation to the presence/absence of a functional p53 protein.

Notably, when we looked at protein expression levels of EpCam, a protein that recently acquired increased interest for its multiple roles in enhancing tumorigenesis [32], and has been reported to be involved in the aggressive phenotype of ATCs [31], we found that PATZ1 expression affects hyper-glycosylation of the protein associated with the EGF treatment of the cells. According to a previous report [44], this could have effects on EpCam stability, with likely consequences on its pro-tumorigenic functionality. Therefore, PATZ1 overexpression in FRO cells affects EpCam expression at both RNA and protein levels, and this may account for the suppressor role of PATZ1 on EMT.

In conclusion, we demonstrated that PATZ1 exerts an oncosuppressor role in thyroid cancer, particularly in the progression to an anaplastic phenotype, through the regulation, at least in part, of p53-target genes *EpCam*, *RhoE* and *Caldesmon*, thus resulting in reduced migration and invasion *in vitro*, as well as MET and reduced tumor growth *in vivo*.

## METHODS

### Tissue collection

Thyroid tissues were collected at the Istituto dei Tumori di Napoli, Italy and the Service d'Anatamo-Pathologie, Centre Hospitalier Lyon Sud, Pierre Benite, France. For each tumor, some fragments were frozen and stored in liquid nitrogen, others were fixed in 4% paraformaldehyde and embedded in paraffin. Informed consent for the scientific use of biological material was obtained from all patients and the work has been approved by the local Ethical Committee.

### RNA extraction and quantitative real time (qRT)-PCR

Total RNA extraction was performed with TRIzol reagent (Invitrogen, Carlsbad, CA), according to the manufacturer's instructions. Reverse transcription was performed according to standard procedures. qRT-PCR analysis was carried out using the Power SYBR Green PCR Master Mix (Applied Biosystems), according to manufacturer's instructions. Primer sequences were as follows: human *PATZ1* (5′-TACATCTGCCAGAGCTGTGG-3′/5′-TGCACCTGC TTGATATGTCC-3′); human *G6PD* (5′-GATCTACC GCATCGACCACT-3′/5′-AGATCCTGTTGGCAAATCT CA-3′); murine *PATZ1* (5′-GAGCTTCCCCGAGCTCAT-3′/5′-CAGATCTCGATGACCGACCT-3′); murine *G6PD* (5′-GAAAGCAGAGTGAGCCCTTC-3′/5′-CATAGGAAT TACGGGCAAAGA-3′); *EpCam* (5′-CCATGTGCTG GTGTGTGAA-3′/5′-TGTGTTTTAGTTCAATGATGATC CA-3′); *fibronectin* (5′-CTGGCCGAAAATACATTGT AAA-3′/5′-CCACAGTCGGGTCAGGAG-3′); *Caldesmon* (5′-GAGCGTCGCAGAGAACTTAGA-3′/5′-TCCTCTG GTAGGCGATTCTTT-3′); *RhoE* (5′-AAAAACTGCGC TGCTCCAT-3′/5′-TCAAAACTGGCCGTGTAATTC-3′).

### Chromatin immunoprecipitation

Chromatin immunoprecipitation (ChIP) was carried out with an acetyl-histone H3 immune precipitation assay kit (Upstate Biotechnology, Lake Placid, NY, USA) according to the manufacturer's instruction, as previously described [45]. Input and immunoprecipitated chromatin were analyzed by PCR for the presence of *EpCam, RhoE and Caldesmon* promoter, choosing regions also including p53 consensus site or putative PATZ1 consensus elements. Antibodies used to immunoprecipitate chromatin were: anti-HA (sc-805; Santa Cruz), control IgG (sc- 2027; Santa Cruz). PCR reactions were performed withAmpliTaq gold DNA polymerase (Perkin-Elmer, Monza, Italy). Primers used were: *EpCam (−218/−65)*: (5′ – ATGGAGACGAAGCACCTGG - 3′ / 5′ - GGGACTGCTCACCTCTGG-3′); *RhoE (−2239/−2183)*: (5′ - TGAGTCCACCAATGAAGCCA - 3′ / 5′ - TATGAGGAAATGCAAGTGACGT - 3′); *Caldesmon (−106/−2): (5′ -* CAGGACAATGCATACCACCG - 3′ / 5′ - TAAAACTCCAGACCGCCCTT - 3′); *Caldesmon (−951/−841): (5′ -* ATGAAGAGTTGGTCGGAGCA - 3′ / 5′ – ATGAAGAGACCCACCACCTG - 3′). PCR products were resolved on a 2% agarose gel and stained with ethidium bromide. Semi-quantitative analysis of the gel bands was performed by ImageJ64 software.

### Histological analysis and immunohistochemistry

Mounted sections (6 μm) were stained with H&E using routine procedures. For immunohistochemistry, sections were deparaffinized, placed in a solution of absolute methanol and 0.3% hydrogen peroxide for 30 min, washed in PBS and incubated overnight at 4°C in a humidified chamber with diluted antibodies. The slides were subsequently incubated with biotinylated goat anti-rabbit IgG for 20 min (Vector Laboratories, Burlingame, CA, USA) and then with premixed reagent ABC (Vector) for 20 min. The immunostaining was performed by incubating the slides in diaminobenzidine solution (DAB-DAKO) for 5 min. After chromogen development, the slides were washed, dehydrated with alcohol and xylene and mounted with cover slips using a permanent mounting medium (Permount). The antibodies used were: anti-PATZ1 [11]; anti-E-cadherin (610181, BD Transduction laboratories). Negative controls were performed by omitting the first antibody. The proportion of cells that were positively stained with the anti-PATZ1 antibody was scored as: – (negative), no positive cells; + (low), < 10% of nuclear positive cells; ++ (moderate), 11–50% of nuclear positive cells; +++ (high), > 50% of nuclear positive cells [22]. At least 20 high-power fields were chosen randomly, and 2,000 cells were counted.

### Protein extraction and western blot analysis

For protein extraction, cells were lysed in lysis buffer containing 1% NP40, 1 mM EDTA, 50 mM Tris-HCl (pH 7.5) and 150 mM NaCl, supplemented with complete protease inhibitors mixture (Roche, Monza, Italy). Total proteins were separated on a 8–10% polyacrylamide-SDS gel electrophoresis and transferred to nitrocellulose membranes (GE Healthcare, Milano, Italy) by electroblotting. Membranes were blocked with 1X TBS, 0.1% Tween-20 with 5% BSA and incubated with antibodies. The antibodies used were as follows: anti-PATZ1 (polyclonal antibody raised against a conserved peptide recognizing all PATZ isoforms of mouse and human origin), anti-EpCam (sc-25308; Santa Cruz), anti-γ-tubulin (sc-17787; Santa Cruz), anti-vinculin (sc-7649; Santa Cruz).

### Cell lines, transfections and plasmids

All human thyroid carcinoma cell lines were cultured in DMEM supplemented with 10% FBS, L-glutamine, and penicillin/streptomycin (GIBCO-BRL) in a 5% CO2 atmosphere. TPC1 and FRO cells were transfected using Neon Transfection System (Invitrogen), whereas BC-PAP cells were transfected using Lipo2000 (Invitrogen), according to manufacturers' instructions. For stable transfections all the cell lines were transfected with PATZ1-EGFP-C2 plasmid carrying human PATZ1 variant 4 cDNA, or with the empty vector pEGFP-C2 (Clontech). Stable transfectants were clonally selected in complete medium containing 1 μg/ml G418 (Life Technologies). pCEFL-HA [23], and HA-PATZ1 plasmids, carrying the human PATZ1 variant 4 cDNA fused to the HA tag, were used in the colony assay.

### Colony assay and growth curves

For colony assay the cells were plated at a density of 90% in 100-mm dishes, transfected with 5 μg of empty vector pCEFL-HA, or HA-PATZ1 plasmid, and supplemented with G418 (Life Technologies) 24 h later. Two weeks after the onset of drug selection, cells were fixed and stained with 0.1% crystal violet in 20% methanol for 30 min, washed with PBS and photographed.

For the growth curves the cells (4 × 10^4^ cells/dish) were plated in a series of 6-cm culture dishes and counted daily for 10 consecutive days through the Bürker chamber. The count was performed in the presence of trypan blue, to assess cell viability.

### Migration and invasion assays

To detect the changed capacity of tumor cell migration, we performed a wound-healing assay. Specifically, cells were digested with 0.25% trypsin and adjusted for a concentration of 5 × 10^5^ cells/ml of cell suspension, and then inoculated into 6-well plates and cultured at 37°C overnight. In the next day, cells were cultured in serum-free medium for 6 h, reached approximately 95–100% confluence, and cell monolayer was wounded by 20 μl tips. The cells were then rinsed twice with culture medium and incubated for 48 h. At 0, 6, 24 and 30 h, cells were photographed under an inverted microscope.

To evaluate tumor cell migration trans-well cell culture chambers were used, according to described procedures (Corning Costar Corp., Cambridge, MA). Briefly, confluent cell monolayers were harvested with trypsin/EDTA, centrifuged at 1.200 rpm for 5 min, re-suspended in medium without serum and plated (3–5 × 10^4^ cells) to the upper chamber of a polycarbonate membrane filter of 8 μm pore size. The lower chamber was filled with complete medium. The cells were then incubated at 37°C in a humidified incubator in 5% CO2 for 24 h and 48 h. Not migrating cells on the upper side of the filter were wiped off and migrating cells on the reverse side of the filter were stained with 0.1% crystal violet in 20% methanol for 30 min, washed with PBS and photographed under light microscope.

The rate of invasion was carried out by using transwell cell culture chambers in the presence of Matrigel (BD Biosciences). The chambers were pretreated with a cold solution containing serum-free medium and Matrigel (diluted 1:4) and left 45 min in incubator at a temperature of 37°C, at which Matrigel polymerizes to produce a biologically active matrix that resembles the basement membrane of mammalian cells. Then the assay was performed as the migratory assay. Stained cells were lysed in SDS and then counted by measuring their absorbance at 595 nm in three independent experiments.

### *In vitro* and *in vivo* tumorigenic assays

Soft agar assays were performed according to the technique described [24]. Colonies larger than background colony size, set with untransformed rat thyroid cells (PC Cl 3), were counted and the results were expressed as number of colonies/plate. *In vivo* tumorigenicity was evaluated by inoculating control- and PATZ1-transfected cells (2 × 10^6^ cells) subcutaneously into the left and right flank, respectively, of seven immunodeficient nude (7 weeks old) Foxn1 nu/nu female mice (Harlan Laboratories). Tumor occurrence was monitored by measuring with calipers at least once every three days. Tumor volume was determined as (length × width)/2. Care and use of the mice were in accordance with institutional guidelines and were approved by the local ethical committee.

### Statistics

Differences among multiple groups of data were analyzed by one-way ANOVA followed by Dunnett's or Tukey's multiple comparisons test. Differences between two groups of data were analyzed by two tailed unpaired *t*-test.

## SUPPLEMENTARY FIGURES AND TABLE


