# Supplementary material for: PATZ1 acts as a tumor suppressor in thyroid cancer via targeting p53-dependent genes involved in EMT and cell migration
Source: Oncotarget. 2015 Jan 20;6(7):5310–23. doi: 10.18632/oncotarget.2776 (PMC4467151; doi:10.18632/oncotarget.2776)
Supplement: Supplementary file 1 [file oncotarget-06-5310-s001.pdf]

## SUPPLEMENTARY FIGURES AND TABLE

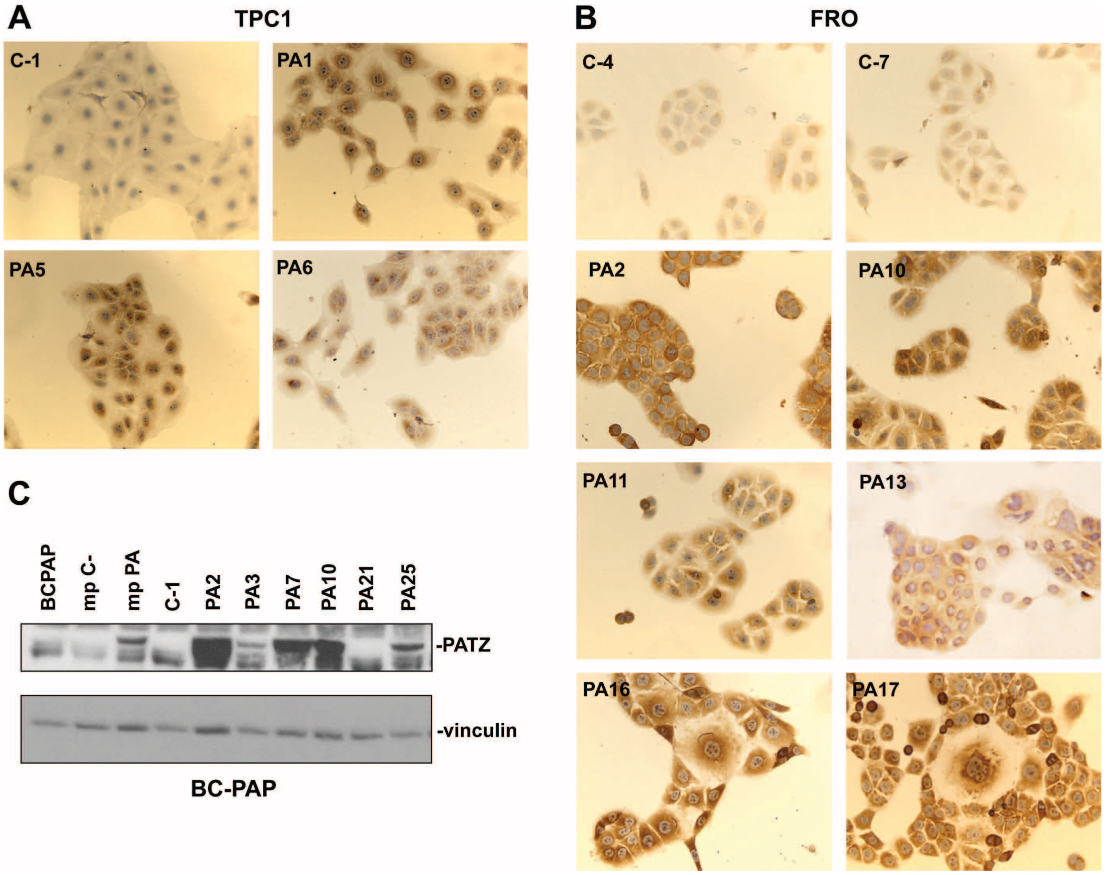


**Supplementary Figure S1: Restoration of PATZ1 expression in TPC1, FRO and BC-PAP cells. (A–B)** Immunocytochemistry for PATZ1 on representative clones of TPC1 (A) and FRO (B) cells transfected with PATZ1 (TPC1/PA1, PA5, PA6; FRO/PA2, PA10, PA11, PA13, PA16, PA17) or empty vector (TPC1/C-1) FRO/C-4, C-7). **(C)** Western blot analysis of PATZ1 in selected clones and mass populations (mp) of BC-PAP cells transfected with PATZ1 (mp PA, PA2, PA3, PA7, PA 10, PA21, PA25) or the empty vector (mp C-, C-1).

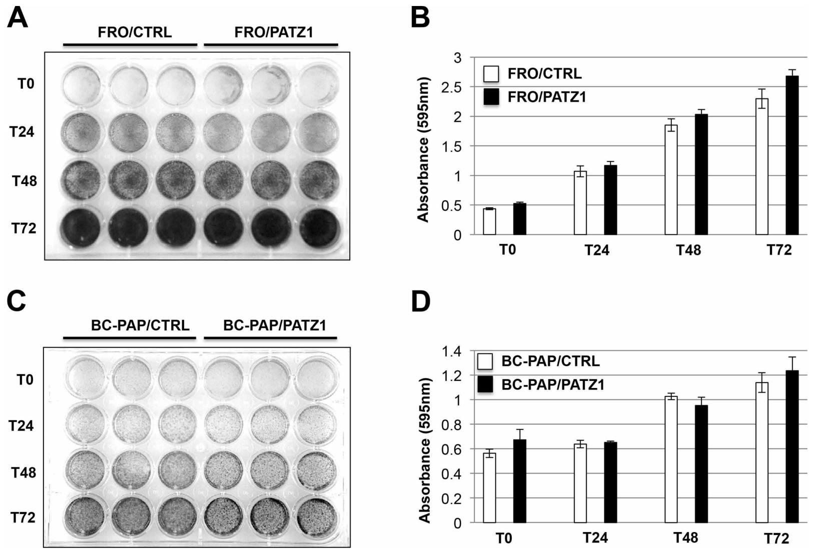


**Supplementary Figure S2: Proliferation rate in FRO- and BC-PAP-transfectants up to 72 h after seeding.** 30.000 cells were seeded in each well of a 24-well plate and stained with crystal violet at the indicated time points after seeding. Each control (CTRL) and PATZ1-transfectant was seeded in triplicate for each time point. **(A, C)** Images of the plates at the end of the experiment. **(B, D)** Mean values ± SE of absorbance at 595 nm of the stained cells.

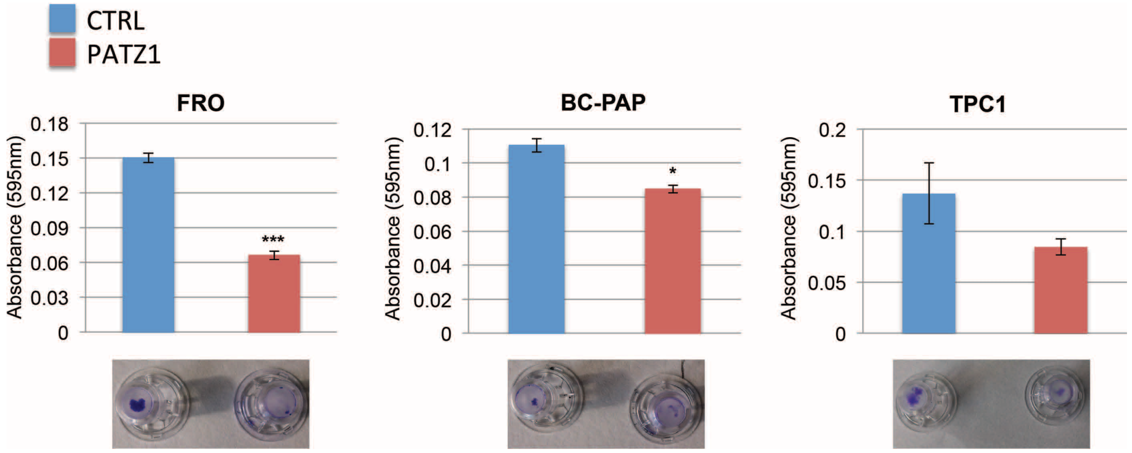


**Supplementary Figure S3: *In vitro* cell invasion based on Boyden Chamber experiments with Matrigel coating.** 48 h after loading of the cells, invading cells were stained with crystal violet. Staining was then dissolved and measured by reading the absorbance of dissolving solution at 595 nm. Inhibition in the number of invading cells in PATZ1-expressing cells compared to controls were observed for all the cell lines. Mean values ± SE of three or four independent experiments performed on a representative clone for each control (CTRL) and PATZ1-transfected (PATZ1) cell line (parental FRO and clone PA17 for FRO; parental BC-PAP and PATZ1-transfected mass population for BC-PAP; parental TPC1 and clone PA6 for TPC1) are reported in the histograms, whereas a representative image of one experiment is shown at the bottom. PATZ1 expression of each clone or mass population is shown in supplementary Figure S1. *, $P < 0.05$; **, $P < 0.01$.

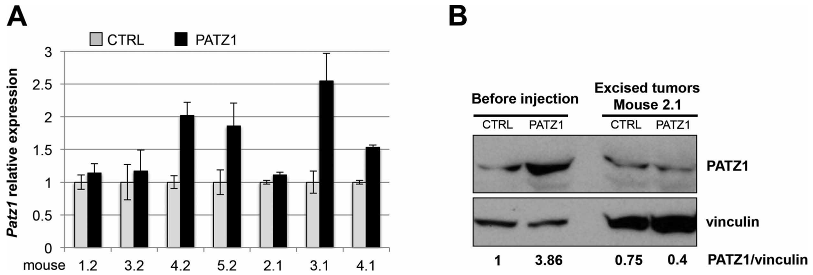


**Supplementary Figure S4: PATZ1 expression in xenografts. (A) qRT-PCR to evaluate expression of PATZ1 mRNA in xenografts excised at the end of the *in vivo* tumorigenic experiment.** Each mouse, whose ID N. is indicated on the bottom, was injected on one flank with empty vector—transfected cells (CTRL) and on the other flank with PATZ1-transfected cells (PATZ1). Mean values ± SE of two or four independent values are reported. **(B)** WB analysis to evaluate PATZ1 protein expression in cells before injection and in a representative excised tumor. Vinculin expression was analyzed as a loading control. The PATZ1/vinculin ratio, evaluated by densitometric analysis, is shown on the bottom.

## Supplementary Table S1: PATZ1 protein expression evaluated by IHC in excised tumors

| Mouse | CTRL tumor | PATZ1 tumor |
|---|---|---|
| **1.2** | Negative | Negative |
| **3.2** | Negative | Negative |
| **4.2** | Negative | Positive areas |
| **5.2** | Negative | Positive areas |
| **2.1** | Negative | Negative |
| **3.1** | Negative | Positive areas |
| **4.1** | Negative | Positive areas |
